# Accidental carbon monoxide poisoning presenting without a history of exposure: A case report

**DOI:** 10.1186/1752-1947-2-118

**Published:** 2008-04-22

**Authors:** Luke Bennetto, Louise Powter, Neil J Scolding

**Affiliations:** 1 Department of Clinical Neurosciences, Frenchay Hospital, North Bristol NHS Trust, Frenchay, Bristol BS16 1LE, UK

## Abstract

**Introduction:**

Carbon monoxide poisoning is easy to diagnose when there is a history of exposure. When the exposure history is absent, or delayed, the diagnosis is more difficult and relies on recognising the importance of multi-system disease. We present a case of accidental carbon monoxide poisoning.

**Case presentation:**

A middle-aged man, who lived alone in his mobile home was found by friends in a confused, incontinent state. Initial signs included respiratory failure, cardiac ischaemia, hypotension, encephalopathy and a rash, whilst subsequent features included rhabdomyolysis, renal failure, amnesia, dysarthria, parkinsonism, peripheral neuropathy, supranuclear gaze palsy and cerebral haemorrhage. Despite numerous investigations including magnetic resonance cerebral imaging, lumbar puncture, skin biopsy, muscle biopsy and electroencephalogram a diagnosis remained elusive. Several weeks after admission, diagnostic breakthrough was achieved when the gradual resolution of the patient's amnesia, encephalopathy and dysarthria allowed an accurate history to be taken for the first time. The patient's last recollection was turning on his gas heating for the first time since the spring. A gas heating engineer found the patient's gas boiler to be in a dangerous state of disrepair and it was immediately decommissioned.

**Conclusion:**

This case highlights several important issues: the bewildering myriad of clinical features of carbon monoxide poisoning, the importance of making the diagnosis even at a late stage and preventing the patient's return to a potentially fatal toxic environment, and the paramount importance of the history in the diagnostic method.

## Introduction

The diagnosis of carbon monoxide poisoning is frequently made obvious by the patients own history; collateral history from attending paramedics or by co-presentation of others who shared a common environment. However patients with carbon monoxide poisoning who present alone and do not, or cannot, give a history of exposure are acutely dependent upon their physicians' ability to recognise an aggressive multi-system presentation for which carbon monoxide poisoning is the only tenable *unifying *diagnosis. We present a case of accidental carbon monoxide poisoning without an early exposure history.

## Case presentation

A 42-year-old man presented with amnesia, pyrexia, hypotension and a rash on his left leg and buttocks. He had been discovered by his friends in a semi-comatose, incontinent condition on the floor of his mobile home. His friends had become concerned when he failed to return their telephone calls for the preceding 48 hours.

Paramedics had been called and found him to be pyrexial, hypotensive, tachypnoeic and tachycardic. His Glasgow Coma Score (GCS) was 7. He had been doubly incontinent. His chest was clear. Pulse oximetry had revealed haemoglobin saturations of 91% on air rising to 96% with oxygen administration. He had a large sacral pressure sore and a rash on his left leg.

On arrival in the accident and emergency department of our hospital he remained confused and disorientated with his GCS having improved to 12, and the tachypnoea, tachycardia and hypotension having resolved. His pulse oximetry had improved to 99% on oxygen. Arterial blood gas examination was normal at this stage, although critically carboxyhaemoglobin levels were not measured. ECG revealed inferolateral T wave inversion. Chest X-ray was normal. He had mild renal failure and a markedly elevated creatinine kinase (CK) level of 12,752 iu/L. Urine toxicology screen was negative. He was treated empirically with antibiotics for a presumed bacterial skin infection of his left leg. He was also treated intravenously with aciclovir, vitamins B1, B2, B6 and nicotinamide. Blood cultures taken prior to antibiotic administration grew a coagulase-negative staphylococcus suspected to be a contaminant. A CT brain scan, and a subsequent MRI brain scan, were both normal. Lumbar puncture revealed <5 white cells but 5810 red cells and a protein of 1.38 g/L. CSF spectroscopy suggested subarachnoid bleeding by revealing the presence of bilirubin. A cerebral angiogram was therefore performed but was normal. Electroencephalograph revealed moderate diffuse cerebral dysfunction consistent with encephalopathy. Extensive blood tests including HIV, anti-GQ1b antibodies, porphyrins, Lyme serology and ammonia levels were all normal.

During the course of the first week of hospitalisation the patient's confusion resolved although he remained amnesic for the two day period preceding his discovery. Similarly his renal failure resolved with intravenous fluids. However neurological examinations during the first week of admission revealed a deteriorating dysarthria, mild bilateral facial weakness, impaired voluntary upgaze, bradykinesia and a mild flaccid tetraparesis with hyporeflexia evolving to areflexia. His CK peaked at 51,825 iu/L four days after admission and remained elevated for a further two weeks. The rash on his leg showed little improvement with antibiotics. Further examination of this lesion revealed a raised firm purple partially bullous plaque that was not typical of cellulitis.

**Figure 1:**
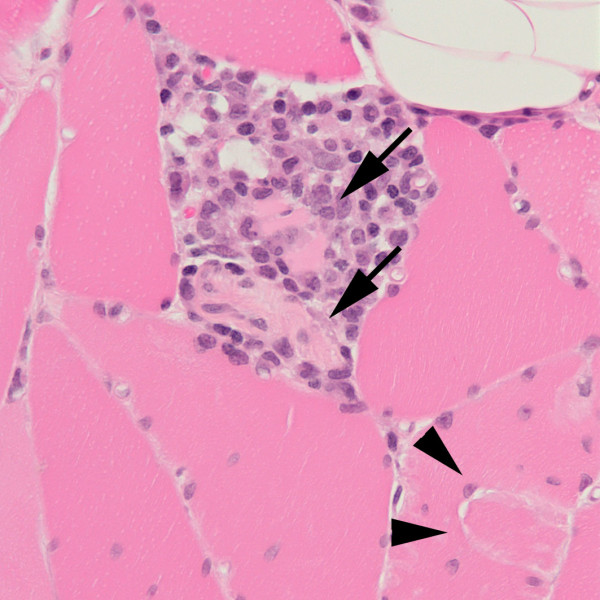
**Section of vastus medialis adjacent to a region of myotendinous insertion (arrowheads).** The figure includes two necrotic fibres (arrows) that are infiltrated by macrophages, with a surrounding aggregate of macrophages and lymphocytes.

**Figure 2:**
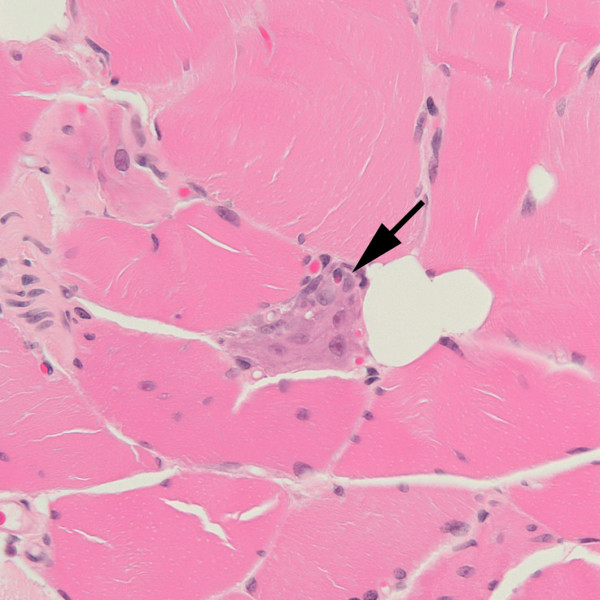
**This section of vastus medialis also near a region of myotendinous insertion, includes a regenerating fibre (arrow) that appears basophilic.** Within the fibre are enlarged nuclei that contain prominent nucleoli.

Because of his progressive neurological problems, further investigations were undertaken. A repeat lumbar puncture revealed an opening pressure of 9.5 cms, protein 1.57 g/l, glucose 3.3 mmol/l (serum 5.8 mmol/l), no white cells, 8 red cells, matched oligoclonal bands, normal cytology and negative spectroscopy. Repeat MRI brain scan remained normal. Muscle biopsy of the right vastus medialis revealed muscle fibre necrosis and regeneration but was otherwise normal (see Figures 1 and 2). Biopsy of the plaque on the left leg revealed marked oedema with a mild perivascular infiltrate suggestive of a purpuric rash. There was no evidence of infection, malignancy or vasculitis. A unifying diagnosis remained elusive.

By the third week after admission the patient began to slowly improve. Partial resolution of his dysarthria, amnesia and encephalopathy aided dialogue and the first person history was obtained for the first time. The patient recalled that on the day he became unwell it had been the first cold day of autumn and he had put his gas heating on. He had last used his heating several months before and due to financial constraints his gas boiler had not been serviced for several years. Turning on the heating was the last clear event he recalled prior to being in hospital. Carbon monoxide poisoning was suspected. An emergency gas engineer found the patient's toxic gas boiler: it was in a dangerous state of disrepair whilst a heavy growth of ivy over the summer had come to further impede ventilation. It was decommissioned and replaced.

## Discussion

Carbon monoxide (CO) is the commonest fatal poison in the United Kingdom [1]. CO is a colourless, odourless gas that is produced by incomplete combustion of hydrocarbons. It is easily absorbed through the lungs and competes with oxygen for binding to haemoglobin. The affinity of haemoglobin for carbon monoxide is 200 to 250 times as great as its affinity for oxygen [2].

Carbon monoxide toxicity is dependant on the concentrations of CO and oxygen in the ambient air and the duration of exposure. At the cellular level damage is probably due to a combination of hypoxia and a direct toxic effect of CO on mitochondrial function. Sources of CO poisoning include vehicle exhausts, poorly ventilated heating systems and inhaled smoke. Whilst deliberate carbon monoxide poisoning rarely cause diagnostic confusion, a substantial minority of carbon monoxide poisoning is accidental. In these cases the confusing array of non-specific clinical features frequently leads to diagnostic error [2] with approximately one third of non-fatal cases believed to be undiagnosed.

Carbon monoxide poisoning has previously been associated with amnesia [3], encephalopathy [4], dysarthria, parkinsonism, peripheral neuropathy [5], bullous skin lesions [6], supranuclear gaze palsy [3], cerebral haemorrhage [7], cardiotoxicity [8] and muscle necrosis with renal failure [9]. In this case the combination of all the above clinical features in the presence of normal cerebral imaging produced considerable clinical confusion that was not relieved by intensive investigation. Ultimately, despite extensive investigation, it was the resolution of amnesia, encephalopathy and dysarthria that allowed the history given by the patient to provide the diagnosis.

Other features of this case are strongly supportive and indeed illustrative of the diagnosis. These include the initial and severe tachypnoea, tachycardia and transient cardiac ischaemia [8] that rapidly resolved with high flow oxygen. Evidence of scattered muscle fibre necrosis in the vastus medialis (see Figures 1 and 2), a muscle not usually associated with typical gravitational rhabdomyolytic pressure necrosis, suggests that the rhabdomyolysis in this case was the result of more than simply being on the floor for two days. Carbon monoxide poisoning is entirely consistent with normal MRI brain imaging [10], although it can also be associated with lesions of the globus pallidus, white matter change and diffuse low density lesions. In this case MRI imaging was performed on a 1.5 tesla scanner and T1, T2, Proton density and Fluid Attenuation Inversion Recovery sequences were used for both scans whilst additional diffusion weighted and magnetic resonance spectroscopy were performed for the second MRI scan. We suggest that the presence of CSF bilirubin in combination with normal cerebral imaging was a result of carbon monoxide induced microscopic intracerebral haemorrhage, a hypothesis supported by previous associations between carbon monoxide poisoning and intracerebral haemorrhage [7].

Carbon monoxide poisoning is a multi-system disease and can cause a confusing constellation of clinical features, precipitating presentation to general practitioners, accident and emergency departments, acute care physicians, general surgeons, neurologists and even psychiatrists. With increasing specialisation within the medical profession the diagnosis may be missed by the specialist who fails to recognise the significance of pathology outside his or her own area of interest.

The benefits of prompt diagnosis are threefold. Firstly recommended therapy, in the form of 100% normobaric oxygen in all cases and hyperbaric oxygen in cases of life threatening poisoning [2] can be instigated. Secondly, as illustrated by this case, unnecessary expensive and painful investigations can be avoided. Thirdly, and perhaps most importantly, the dire consequences of discharging a patient home to, or allowing others access to [10] a potentially fatal environment can be avoided.

## Conclusion

This case illustrates several important issues: the bewildering myriad of clinical features of carbon monoxide poisoning, the importance of making the diagnosis even at a late stage and preventing the patient's return to a potentially fatal toxic environment, and the paramount importance of the history in the diagnostic method.

## Competing interests

The authors declare that they have no competing interests.

## Authors' contributions

LB drafted the manuscript. NJS first considered the diagnosis and in conjunction with LP helped revise the manuscript. All authors were both involved directly in the patient's care and read and approved the final manuscript.

## Consent

Written informed consent was obtained from the patient for publication of this case report and any accompanying images. A copy of the written consent is available for review by the Editor-in-Chief of this journal.
